# Concepts of Confidence in Tendency Survey Research: An Assessment with Multi-group Confirmatory Factor Analysis

**DOI:** 10.1007/s11205-014-0736-1

**Published:** 2014-09-05

**Authors:** Piotr Białowolski

**Affiliations:** Institute of Statistics and Demography, Collegium of Economic Analyses, Warsaw School of Economics, Ul. Madalińskiego 6/8, 02-513 Warsaw, Poland

**Keywords:** Confidence indicators, Formative and reflective approach, Multi-group confirmatory factor analysis, Tendency surveys

## Abstract

In this paper, we investigate the link between the formal definition of confidence in tendency surveys and its measurement. We advocate for the use of reflective measures in an assessment of the confidence level in both consumer and industrial indicators. Based on the data from Poland’s tendency survey research, we use a multi-group confirmatory factor analytical approach to demonstrate that the set of indicators proposed by the European Commission methodology that is currently used might be not appropriate to measure the concept of confidence consistently, both within and between periods. The conclusion is true for the confidence indicator in the area of consumer tendency surveys and for the tendency survey in the manufacturing industry. We search for possible amendments that help either to find the sources of instability for the indicators proposed by the guidelines of the European Commission or to select a different set of indicators for the concept of confidence. However, we determine that the differences between the newly proposed indicator that describe industrial confidence and the indicators based on the European Commission methodology are small in terms of correlations and predictive validity.

## Introduction

The results of tendency surveys are usually summarised every period in a single number—the confidence index. Currently, the European Commission (2006) tracks changes in European countries’ economies on a monthly basis using confidence indicators in five sectors, with industrial and consumer confidence indicators among the most important. The confidence indicators have been developed to track most accurately the changes in their reference series, which is gross value added in the case of the industrial confidence indicator and private consumption expenditures in the case of the consumer confidence indicator (European Commission 2006, p. 24). In a commonly applied approach to confidence measurement, indices are calculated every period for every sector as a simple (or weighted) average of balances calculated for each of the predetermined questions. Balances, in turn, represent differences between the share of “positive” and “negative” answers for each question (European Commission 2006). Thus, the aggregate information is obtained without investigating interactions between answers to questions on an individual (household, company) level. With such an approach, a few crucial questions concerning validity and reliability of consumer confidence are left unanswered:

1. Do we really, with the chosen set of questions, capture a concept that can be associated with confidence?
2. How should we measure the concept of confidence—is it formative or reflective?
3. Is the set of confidence indicators (questions) coherent in each period of analysis?
4. Does the understanding of questions and the mode of answering in different time periods remain constant?

Leaving these questions aside might lead to misinterpretations of the confidence indicators’ values, as they might be neither reliable nor valid. A lack of coherence in the set of questions can manifest itself in two ways. First, the values of the index of confidence may reflect only unidimensional projections of a phenomenon that is multidimensional in reality. Second, the meaning of the concept might evolve between periods or the concept might even completely lose its initial sense. Violation in one of the areas can lead to significant problems with interpretability of the index. Unidimensionality is crucial for establishing that there is a single concept behind the answers to the selected questions (OECD-JRC 2005).^1^ Without verification of intertemporal consistency, the comparisons of values of the obtained confidence index might be unjustified due to a lack of constant meaning throughout the timeframe of analysis (Coertjens et al. 2012; Davidov 2008).

A need to assess the measurement issues in tendency surveys was clearly stated by Lemmens et al. (2007) and Nahuis and Jansen (2004). Nevertheless, to the best of our knowledge, there have not been any attempts to put it into practice to date. Thus, the main goals of this paper are as follows. First, we evaluate the possibility of developing a unidimensional scale to measure (1) consumer confidence and (2) industrial confidence with the set of questions from the consumer tendency survey and business (industrial) tendency survey, respectively, proposed by the European Commission methodology (European Commission 2006). Second, we propose alternative measurement solutions when the desired measurement quality cannot be reached.^2^ Third, we want to raise awareness about the importance of measurement equivalence in tendency surveys. Finally, we aim to start a discussion about reliable and valid measurement in the field.

In this paper, we feature three innovative points. First, this article is the only article to date that attempts to propose a formal definition of confidence in tendency surveys and the way in which it should be measured. Second, this article is the first check of the validity of the commonly used indicators of industrial and consumer confidence. Third, it is also the first approach to search for inconsistencies and provide amendments in the confidence indicators used in industrial and consumer tendency surveys.

The article is organised as follows. In Sect. 2, we describe the concept of confidence and develop an approach to its measurement in both consumer and industrial tendency surveys. In Sect. 3, we present a methodology for the measurement and invariance assessment of concepts in tendency survey research. In Sect. 4, we describe our data and verify whether the confidence indices for the consumer and manufacturing (industrial) sectors based on the results of surveys conducted at the Research Institute for Economic Development in Poland can be presented as a single factor with a standard set of items. We also search for solutions to address inconsistencies.

## Conceptualisation and Operationalisation of the Concept of Confidence

The main goal of constructing composite measures is to provide a compromise between the usually large amount of information gathered in surveys and a straightforward interpretation of a single number (OECD-JRC 2005; Saltelli 2007). With a single number, the general public gains information on the research outcome without being drawn into scientific nuances of the whole survey. However, one should remember that there are also disadvantages of composite indicators. In a formative approach, when unidemensionality is not ascertained, there is (1) loss of the information content and (2) the possibility that misinterpretation of the results might occur. Problems associated with these two issues should be addressed and mitigated during the construction process of a composite indicator—by assuring that the questions used for the construction of the indicator directly match the concept that is being measured and by properly selecting a method by which the composite index is calculated.

To ensure that the concept directly matches our intentions, it should first be established whether it belongs to the set of concepts-by-intuition or concepts-by-postulation (Saris and Gallhofer 2007, p. 15). In line with Saris and Gallhofer’s presentation, concepts-by-intuition refer to simple phenomena that we are able to capture and distinguish with our senses, such as colours (green, blue) or tastes (salty, sweet), etc. More complex phenomena that gain their meaning from theory are in the scope of concepts-by-postulation. Their meaning can be derived from other concepts that we already understand.

### The Concept of Confidence: Definition and Conceptualisation

In tendency surveys, the most commonly used composite scores are confidence indicators (European Commission 2006). The need to use composite scores instead of single indicators for assessment of consumer confidence has been long justified by better forecasting performance of such measures (see, e.g., Shapiro and Angevine 1969). Although the consumer and industrial confidence indicators have been used in many studies mostly as a forecasting tool (Adams 1964; Bialowolski et al. 2014; Carroll et al. 1994; Curtin 1982; Golinelli and Parigi 2004; Kumar et al. 1995; Paradiso et al. 2014, among others), there has been almost no debate on their meaning and consistency.^3^ Apart from forecasting, there are only minor exceptions in which the confidence indicators have been analysed in connection with their components (Jansen and Nahuis 2003; Ramalho et al. 2011). The choice of questions for confidence indicators also lacks proper justification. Mueller (1963, p. 901) states only that the questions serving as items in the American consumer sentiment indicator^4^ were chosen because “they relate to different important aspects of consumer sentiment and because they have been asked at least since 1952”. Nevertheless, some general guidelines concerning the indicator of confidence can be found in the literature. Golinelli & Parigi (2004) claim that, given the rational expectations hypothesis, the indicator has to have additional information if it is defined as an expected value of macroeconomic variables. They also point that consumer sentiment (confidence) is more general concept that cannot be summarised only on the basis of some macroeconomic variables. Vuchelen (2004) states that consumer sentiment is not well understood and there is no agreement on its information content. Additionally, he adds that confidence might be associated with unobserved variables.

Conceptualizing the notion of confidence in tendency surveys as either a concept-by-intuition or a concept-by-postulation should also be supported by a formal definition of the term “confidence”. According to the Merriam-Webster Dictionary, confidence can be described as “faith or belief that one will act in a right, proper, or effective way” or “the quality or state of being certain” (underlined by the author).^5^ According to the Oxford Dictionary website, confidence is “the feeling or belief that one can have faith in or rely on someone or something”.^6^ These three definitions combined with literature developments and the main objective of tendency surveys, which is to provide useful information for the short-term forecasting (European Commission 2006), lead us to a working definition of confidence measured in tendency surveys: “Confidence is the level of certainty that the economic processes will develop in a positive direction, i.e., result in higher level of production, GDP or consumption”.

With a formal definition in hand, a conceptualisation of confidence is required, i.e., establishing a relationship between it and the meaning of the measurement of confidence. We assume that there is a representation of the concept of confidence in responses to a survey, which implies that responses to the survey questions are driven by confidence in the general situation of the economy. It does not undermine the use of responses regarding an individual (household, company) situation as they might also be driven by common confidence, but it indicates that the concept of confidence can be described as a concept-by-postulation, and thus, its conceptualisation is not trivial.

Although the link between the formal definition of confidence and the indicators was not established in the European Commission guidelines (European Commission 2006), there is a commonly used group of questions that serve as confidence indicators for business (industrial) confidence and the group of consumer confidence items. Thus, their validity can be verified. Consumer confidence indicators normally comprise the questions that refer to the future financial situation of the surveyed household (FS.F), future general economic situation of the country (GES.F), future situation on the labour market (UNEMP.F) and the future situation concerning the ability of the household to save (SAV.F).^7^ The balances for each question are computed and subsequently used to calculate the confidence indicator as a simple average. With respect to the business (industrial) confidence indicator, the original selection of variables comprises order books (IND.ORD.S), current stocks of finished goods (IND.STOCK.S) and the forecasted level of production (IND.PROD.F).

Each of these two groups of items (questions), at least at first glance, seems to be internally heterogeneous. With respect to consumer confidence, although all indicators refer to the future, it is expected that the confidence should materialise not only in the area of household position (questions FS.F and SAV.F) but also in the area of the future situation of the general economy (GES.F and UNEMP.F).^8^ With respect to business (industrial) confidence, the controversy in the composition of the index might result from a mixture of indicators that assess the current and future state of affairs in a company but also leading—IND.ORD.S—and lagging—IND.STOCK.S—character with respect to the business cycle (Zarnowitz 1992).

### Measuring Confidence

Although the current state of the art in constructing confidence indices in tendency surveys relies solely on a formative approach, there have been no arguments provided for its superiority over a reflective approach. There is a broad literature on the differences between formative and reflective measurement in science (Baxter 2009; Coltman et al. 2008; Diamantopoulos 2010; Wilcox et al. 2008), but the issue of measurement has gained very little attention in tendency surveys. Although, there are arguments against the current approach, which are best summarised by Pickering et al. (1973) in the following statement “using a simple summation of responses (…) fails to take account of the interrelationship between the variables measured and their differing importance in the overall consumer decision-making process”, a judgement was never made as to whether the concept of confidence should be treated as a formative or a reflective one.^9^

It should be noted that the concepts are usually not intrinsically related to a single, proper method. For instance, Wilcox et al. (2008) present an example when the same concept—coercive power—can be measured with both the formative and reflective approach. They show, however, that the difference in the measurement approach can be associated with orientation in the time of the construct of interest. If researchers are interested in the coercive power exercised by suppliers in the past, they can treat the index of coercive power as a formative one because each action from the set of possibilities (delay delivery, delay warranty claims, take legal actions, charge higher prices, and deliver unwanted products) results in a deterioration of the situation of the recipient. Thus, there is no need for correlations between the variables necessary in the reflective measurement (Brown 2006). However, in an assessment of the coercive power oriented towards future, it would be much more appropriate to use the reflective approach, as each of the single actions undertaken by a supplier, if known in advance, can be mitigated. Thus, only an ability to take the set of actions altogether by the supplier can be perceived as coercive power.

There are advantages to using formative indicators in tendency surveys: they are easier to calculate, they have a long history, and in many studies, they have proven to be a significant predictor of economic development. However, by analogy to the example mentioned above, it can be advocated that the concept of confidence is also measured with the intention to provide information concerning the future development of the economy, which favours the reflective approach. Let us further expand the line of argument in favour of the reflective approach with respect to the measurement of economic confidence. If the higher level of confidence is present, it should have an impact on all economic areas and not be limited to some indicators only, which strongly supports measurement based on correlations. As confidence by definition is designed to predict future developments, following Wilcox et al. (2008), there are more arguments in favour of the reflective approach, which is associated with co-movements of confidence items. Taking into account the above arguments, the aggregation method based on a factor analytical approach rather than a simple averaging of balances is proposed.^10^

In the field of business tendency surveys, there is a considerable ambiguity regarding the meaning of a factor analytical approach. Factor models rely on common information provided by data (Brown 2006; Hurley et al. 1997). With respect to business and consumer survey data, factors in the “common factor” models are associated with fluctuations at the level of aggregate data, which can be summarised as follows: the common factor explains the changes in aggregated answers to questions (see, e.g., Costantini 2013; Gayer and Genet 2006).^11^ This approach, although significant, is not oriented towards the verification of whether a common factor exists at the micro (respondent/household) level and whether it has been stable over time. The factor analysis models based on individual data solve the problem of comparability in time. They enable to check for measurement invariance, which is necessary to state the equal meaning of a concept across groups (Byrne et al. 1989). The groups can be understood differently and can consist of different countries (Byrne and van de Vijver 2010; De Jong et al. 2007; Raijman et al. 2008; Steenkamp and Baumgartner 1998), different periods (Białowolski and Węziak-Białowolska 2013; Coertjens et al. 2012; Davidov 2008; Vandenberg and Lance 2000) or different respondent characteristics (Horn and Mcardle 1992; Raykov et al. 2012).^12^ In the case of tendency surveys, groups are defined as different periods.

The common practice to assess the comparability of a latent concept with MGCFA is to check for the existence of measurement invariance (Brown 2006; Hu and Bentler 1998, 1999; Steinmetz et al. 2007). If established, measurement invariance ensures that the concept is comparable between groups. An additional feature of MGCFA is that the items (questions) that are correlated with the common variance of items (communality) receive much larger factor loadings, and those that are weakly related to communality receive low factor loadings.

Taking into account all of the arguments, namely, the formal definition of the concept of confidence, its future-oriented nature that implies correlations between its indicators (concept-by-postulation), and the advantages associated with the use of micro-level data, MGCFA is subsequently used for measurement of confidence.^13^

## Model Assessment with Multi-group Confirmatory Factor Analysis

### The Measurement Model

MGCFA is a factor analytical approach that accounts for the intertemporal structure of respondent answers. With indicators measured on a categorical scale, the final model was estimated by diagonal weighted least squares while test statistics were computed with the full weigh matrix (WLSMV option in the Mplus program). This method is suitable for items with categorical responses (all tendency survey questions are measured on a categorical scale). Based on the discussion conducted in Sect. 2, the confidence indicator is a latent construct that is operationalised by a set of proxies (questions), which are designed to be its indicators. The formal structure of the estimated model in the case of *N* proxies (questions), one latent variable operationalising confidence and *T* time periods can be given by the following:

1 $$ \forall_{t \in T} {\mathbf{q}}^{t} = {\varvec{\upgamma}}_{1}^{t} \cdot Confidence^{t} + {\varvec{\upvarepsilon}}^{t} , $$

where, in all time periods, ${\mathbf{q}}^{t}$ is the *N* × 1 vector of question answers, ${\varvec{\upgamma}}_{1}^{t}$ is the *N* × 1 vector of the factor loadings for the confidence concept, and ${\varvec{\upvarepsilon}}^{t}$ is the *N* × 1 vector of measurement errors. In this specification, to ensure the identification of the model, one element of the ${\varvec{\upgamma}}^{t}$ vector (factor loading) is set to 1.^14^ Additionally, $E\left( {{\varvec{\upvarepsilon}}^{t} } \right) = {\mathbf{0}}$ and $\forall_{t \in 1..T,p,q \in 1..N,p \ne q} \text{cov} \left( {{\varvec{\upvarepsilon}}_{p}^{t} ,{\varvec{\upvarepsilon}}_{q}^{t} } \right) = 0$. Because it is assumed that the answers to all of the questions are measured on a categorical scale, thresholds indicating a switch between one category and another are estimated, implying that for the i-th respondent, the scoring on the latent variable $Confidence_{i}^{{t}} {^{*}}$ (question answers) is subject to the following constraint

2 $$ \forall _{{t \in 1 \ldots T,p \in 1 \ldots N}} q_{p}^{t}  = m\quad if{\mkern 1mu} {\mkern 1mu} v_{{p,m - 1}}^{t} {\text{  < }}{\mathbf{\gamma }}_{{1,p}}^{t} Confidence_{i}^{{t}} {^{*}} + \varepsilon _{p}^{t} {\text{ }} < v_{{p,m}}^{t} $$

In Eq. (2), m stands for an answer category in the p-th categorical indicator variable, which can have a value ranging from 0 to *M*_*p,*_ 15 *v*_*p*,*m*_^*t*^ represents the m-th estimated threshold for the p-th categorical latent variable,^16^ ${\varvec{\upgamma}}_{1,p}^{t}$ and *ɛ*_*p*_^*t*^ represent the estimated factor loadings and error terms, respectively, which are associated with the p-th categorical response variable in period t.

### Testing for Measurement Invariance of Confidence Indicators

The concept of measurement invariance plays a crucial role in establishing comparability between groups of latent concepts of interest. Because in our case the concept of confidence should be comparable between periods, assuring measurement invariance is crucial for the analysis. Without any additional assumptions, models specified with (1–2) do not allow for time comparisons of the latent variable mean of the confidence concept. Good fit of such a model implies the existence of configural invariance only, which might be used to state that at each time-point there was some unidimensional concept behind the data. To check for the possibility of comparisons between time points of the mean of the latent concepts, the estimated multi-period measurement model must satisfy the condition of at least partial scalar invariance (Byrne et al. 1989; Davidov 2008; Meredith and Teresi 2006; Meredith 1993; Muthen and Asparouhov 2002; Steenkamp and Baumgartner 1998), which implies that at least two factor loadings and at least two sets of thresholds (for the corresponding variables) are set equal between periods.

In the factor analytical approach based on the micro data, the assessment of the measurement invariance might be based on goodness-of-fit statistics. The most frequently used are the Comparative Fit Index (CFI), the Tucker–Lewis Index (TLI), the Root Mean Square Error of Approximation (RMSEA) and the Standardised Root Mean Square Residuals (SMRM). Certain rules were developed for each of these descriptive fit statistics. These guidelines are mostly based on simulations (Chou and Bentler 1995; Kaplan 2009). With respect to CFI and TLI indexes, it is usually assumed that their values should be above 0.9 to judge the model as acceptable (Hox 2002, p. 239; Hu and Bentler 1999). The values of RMSEA and SMRM should be below 0.08 (Browne and Cudeck 1993).^17^

In this paper, we assess the goodness-of-fit of a model based on CFI, TLI and RMSEA.^18^ We assume that to accept a model, all of the goodness-of-fit statistics should lie within an acceptable range. An acceptable fit must be obtained for the model with at least partial measurement invariance.

Evaluation of the model fit was conducted according to the following strategy. The analysis starts from the model with configural invariance. If it is achieved, full measurement invariance is verified, but if the acceptable fit based on descriptive fit statistics is not obtained, the factor loadings and thresholds are sequentially relaxed. This procedure is conducted until an acceptable fit is obtained or the number of indicators in the model with relaxed factor loadings and thresholds reaches N-2*F, where F stands for the number of factors. If an acceptable fit is not possible, the procedure stops without establishing partial measurement invariance. However, if at least partial scalar invariance cannot be obtained with the current set of questions, a different solution is proposed to obtain a valid index of confidence.

## Validity Assessment of Confidence Indicators in Consumer and Business Surveys

With MGCFA, the validity of the set of indicators in consumer and business tendency surveys is assessed. It is checked whether the current set of indicators is driven by a common cause, which can be associated with confidence. Subsequently, it is assessed whether comparisons of confidence can be performed between periods. The assessment process is conducted separately for business (industrial) confidence and for confidence among consumers.

### Data and Strategy for Handling Measurement Non-invariance

**Fig. 1:**
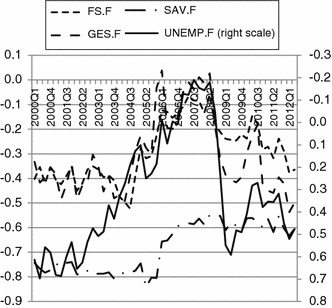
Balances of answers to the questions (components) of the consumer confidence indicator. *Source*: Own calculations in IBM SPSS Statistics 21

The analyses conducted in this article are based on the data from the Research Institute for Economic Development (RIED) from the Warsaw School of Economics. Two sets of quarterly data were used. One set comprised the consumer tendency survey (The State of the Households Survey), which has been conducted at RIED starting from the first quarter of 1996 on a quarterly basis. Initially, the survey was conducted via a questionnaire attached to a newspaper. Since 2000, it has been conducted via a post questionnaire based on a representative sample of Polish households. Due to different methodologies and possible method bias in the data, only data starting from year 2000 were used in the final analyses. Each quarter approximately 3,000 Polish households received the questionnaire. The response rate oscillated approximately 20 %. Within the analysed period (1st quarter 2000–2nd quarter 2012) the average number of responses were 636 with a maximum 1,179 in the 1st quarter 2001 and a minimum of 371 in the 3rd quarter 2006. The basic statistics for the data are presented in the form of balances in Fig. 1.

It can be observed that at the level of aggregates all balances are strongly correlated, with the only exception being correlation between household savings and both questions referring to the general economy (GES.F, UNEMP.F). The strong co-movement of aggregates is only the first step, however, and further analyses are oriented to establishing whether there is a common cause—confidence, which can be compared between time points. Due to strong interrelation between the initial set of items in the consumer confidence case, the adopted procedure of handling measurement non-invariance was oriented on showing the dimensionality of the current consumer confidence indicator.

**Fig. 2:**
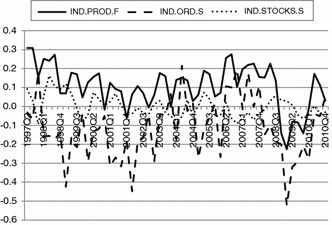
Balances of answers to the questions (components) of the industrial confidence indicator. *Source*: Own calculations in IBM SPSS Statistics 21

The second data source is an industrial tendency survey conducted among manufacturing firms in Poland in line with the harmonised European Commission questionnaire since the second quarter 1997. The survey is conducted on monthly basis; however, for the purposes of the analysis, only information from the first month of each quarter was used.^19^ The initial sample comprised of a randomly selected group of Polish enterprises from the database of manufacturing firms from the Central Statistical Office. The time span of the analysis covered the period from the beginning of the survey to the 4th quarter 2010, which is 55 quarters. The average number of responses was 559 with a maximum equal to 1,043 in the 2nd quarter 1997 and minimum equal to 333 in the 3rd quarter 2007. The average response rate was approximately 30 %. The problem of missing data is also limited with as little as 0.8 % of missing data with respect to price policy (IND.PRA.S) and maximum of 9.8 % with respect to the forecasted capacity utilisation (IND.CAP.F). The balances for the initial components of the industrial confidence indicator are presented below (Fig. 2).

Contrary to the situation observed in the consumer tendency survey, based on aggregates it is hard to find a justification for a common force, which would be associated with confidence. Especially the time series of responses to the question regarding the stocks is loosely related to the other two and the correlation coefficient has a different sign with respect to production forecasts (IND.PROD.F) and the state of orders (IND.ORD.S). The aforementioned low correlation implies that the question regarding the stocks provides noise to the variation of the scores of the composite of industrial confidence. On the other hand, the negative correlation suggests the existence of trade-offs between indicators, implying that an increase in one indicator is likely to decrease the composite. Both issues are considerably troublesome from the methodological point of view, questioning not only the existence of a common factor driving the data but also being strong indication for choosing different set of indicators (Athanasoglou et al. 2014; Saisana and Weziak-Bialowolska 2013). Due to considerable conceptual problems with the initial set of indicators in the industrial tendency survey, the adopted procedure for handling measurement non-invariance was different from in the consumer confidence case. Proposed solutions were of an exploratory nature and the search for the best indicator was based on estimating all of the possible models containing the set of three indicators from the tendency survey in manufacturing industry.^20^ The goal of these estimations was to show that there is a possibility to obtain a good, consistent (but based only on three questions, the lowest possible number) indicator of a concept driving the responses to questions provided by companies.

### Consumer Confidence

To assess the existence of a common factor behind the answers to the original set of consumer confidence items, we performed a check of the one factor solution explaining the variation in the dataset, starting from the model with configural invariance.^21^ Although factor loadings for all indicators were salient above 0.4 (Brown 2006; Matsunaga 2011; Osborne and Costello 2004), which shows that all questions are related to communality (common variance) in all periods, the fit of this model was below the acceptable level (CFI = 0.957, TLI = 0.913, RMSEA = 0.125). This, in turn, points to poor validity of the consumer confidence indicator and is sufficient to refrain from testing higher levels of measurement invariance in this specification. As an alternative, we checked for the possibility of obtaining a two-factor solution with questions related to the situation of the household explained by one factor (CCI_HH) and questions referring to the general economic situation explained by the second factor (CCI_GS). The fit of such a model with configural measurement invariance properties assumed was satisfactory (Table 1, model No. 1). Additionally we checked two other alternatives of grouping four items by two into two factors (Table 1, model No. 2 and 3) but we also performed a check of full measurement invariance with respect to the selected specification (Table 1, model No. 4).

**Table 1:** Two factor models of consumer confidence—configural and scalar invariance

Model	Factor 1	Factor 2	CFI	TLI	RMSEA
No.					
Two factor solution—configural invariance					
1	FS.F SAV.F	GES.F UNEMP.F	.985	.969	.075
2	FS.F GES.F	UNEMP.F SAV.F	.942	.883	.145
3	FS.F UNEMP.F	GES.F SAV.F	.976	.951	.094
Two factor solution—scalar invariance					
4	FS.F SAV.F	GES.F UNEMP.F	.831	.871	.152
Largest sub-sample with scalar invariance (1st quarter 2004–1st quarter 2008)					
5	FS.F SAV.F	GES.F UNEMP.F	.948	.959	.079

*Source*: Own calculations in Mplus

Among the first three analysed solutions, only model No. 1 proved to be sufficiently well fitted—within the boundaries suggested by the literature with respect to all of the descriptive fit statistics. Unfortunately, the model with full measurement invariance was not fitted well,^22^ which implied that comparisons of latent variable means were not allowed in the whole analysed period. Therefore, based on the information provided by contribution of the Chi square statistics in each period, the longest subsample (sub-period) characterised by consistent measurement with scalar invariance was established. In this way model adequacy in the period 1st quarter 2004 and 1st quarter 2008 was confirmed (Table 1, model No. 5). The estimation of the model (model No. 5) led to the following results:

3 $$ \left\{ {\begin{array}{*{20}l}    {FS.F^{t}  = 1 \cdot CCI\_HH^{t}  + \varepsilon _{1}^{t} \quad {\text{thresholds}}: - 1.628, - 0.464,0.505} \hfill  \\    {SAV.F^{t}  = \mathop {0.766}\limits_{{(0.044)}}  \cdot CCI\_HH^{t}  + \varepsilon _{2}^{t} \quad {\text{thresholds}}: - 2.099, - 0.336} \hfill  \\    {GES.F^{t}  = 1 \cdot CCI\_GS^{t}  + \varepsilon _{3}^{t} \quad {\text{thresholds}}: - 1.356, - 0.572,0.313} \hfill  \\    {UNEMP.F^{t}  =  - \mathop {0.885}\limits_{{(0.043)}}  \cdot CCI\_GS^{t}  + \varepsilon _{4}^{t} \quad {\text{thresholds}}: - 0.839,0.090,1.042} \hfill  \\    {corr(CCI\_HH,CCI\_GS) = \rho ^{t} } \hfill  \\   \end{array} } \right. $$

**Fig. 3:**
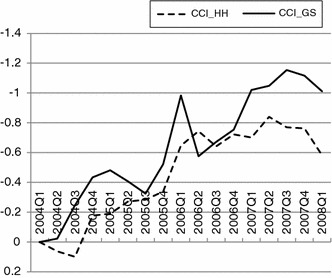
Consumer confidence with respect to household (CCI_HH) and general economic (CCI_GS) situation in the period 1st quarter 2004–1st quarter 2008. *Source*: Own calculations in Mplus 6.1

The model (3) shows that a one point increase in the value of CCI_HH results in a one point increase with regard to financial situation forecasts, 0.766 points increase on the scale of question regarding savings forecasts, while a similar increase in the value of CCI_GS results in a one point increase in response to the general economic situation forecast and 0.885 points decline in the question regarding forecast of the unemployment rate. Additionally, the correlation coefficient between the two dimensions of confidence (CCI_HH and CCI_GS), estimated for each period separately, shows that the co-movement of respondents’ responses in the two analysed dimensions is strong but varies over time. The values of correlation coefficients range from 0.564 in the 3rd quarter 2007 to 0.965 in the 3rd quarter 2005. Period specific averages of the two dimensions of consumer confidence are presented in Fig. 3.

The results suggest that confidence with respect to the household situation is closely related to the confidence with respect to the general economic situation, which is confirmed by correlation of the two time-series equal to 0.895.

Interesting conclusions can be drawn from the analysis of time span for which a consistent measurement could have been obtained. Before 2004, the Polish economy underwent an economic slowdown with average growth rate in the period 2000–2003 at the level of 2.7 %. In the period from the 1st quarter 2004 to the 1st quarter 2008, when consistent measurement was obtained, average GDP growth rate exceeded 5.5 %. The period of consistent measurement ends just before the onset of the financial crisis, when the growth rate suddenly declined.

The analysis conducted in this paragraph indicates that for the data from the Poland State of the Household Survey the responses to the questions comprising the standard set of indicators of consumer confidence were not driven by a single concept that could be associated with confidence. It thus implies that valid measurement of a single one-dimensional confidence concept cannot be achieved with this set of indicators because for a one-dimensional model even configural invariance could not have been achieved. It appears that a probable cause is the two-dimensional nature of the set of indicators that is currently used. However, even in the case of a two-dimensional concept, it was not possible to show a constant measurement—associated with full measurement invariance—for the whole time span of the analysis. Only after was the time span of analysis was limited, a consistent measurement could have been ascertained.

The two dimensions of consumer confidence in the obtained measurement model are clearly interrelated, which shows that better assessment in household dimension is likely to correlate with better assessment of the general economy. Nevertheless, the two-dimensional nature implies confidence that the economic processes will develop in a positive direction and translate differently into expectations regarding the general economic situation and unemployment and differently into expectations regarding the household financial situation and savings.

### Industrial Confidence

The validity assessment was also performed with respect to industrial confidence. The set of indicators proposed by the European Commission guidelines (2006) is based on three indicators: orders (IND.ORD.S), current stocks of finished goods (IND.STOCK.S) and the forecasted level of production (IND.PROD.F). The initial check of the validity of the industrial confidence indicator with assessment of the full scalar measurement invariance, in which all three indicators serve as proxies for consumer confidence, did not provide satisfactory results (CFI = 0.757, TLI = 0.815, RMSEA = 0.096).^23^ With partial measurement-invariant models, when factor loading and thresholds were released for one item at a time, only a slight improvement was gained. The models with different period-variant items proved to be either impossible to estimate due to convergence problems or characterised by a mediocre gain in the model fit. The best estimate (CFI = 0.821, TLI = 0.727 and RMSEA = 0.116), though not sufficient, was obtained in the model with partial scalar measurement invariance with relaxed factor loading and thresholds associated with the state of stocks (IND.STOCK.S). However, even in this model the final factor loadings were not salient.

Thus, it might be stated that the three indicators are weakly associated with any single factor, and therefore, at least conceptually, they do not reflect any specific concept and particularly do not reflect the concept of confidence. This conclusion is strongly consistent with our findings based on the correlations between the balance-based indicators presented in Fig. 2 and requires a search for a different set of industrial confidence indicators.

It might be noted that in the questionnaire of the survey in manufacturing 16 out of 18 questions are associated with current company situation and the remaining two refer to the general economic situation (see Appendix 2). Thus, following the results obtained with respect to consumer confidence, it might be expected that if there is a representation of confidence in the survey, it is likely to be reflected in the set of questions from one realm only. However, a form of exploratory analysis was performed to find a set consisting of three (the lowest possible number for a composite indicator in MGCFA) out of 18 questions from the survey that fit the full scalar measurement invariant model well.

With the Mplus Automation package, which allows to run Mplus under R, there were 816 models estimated, which comprised all possible combinations of three element sets out of the eighteen questions with an assumption of full measurement invariance. There were only nine specifications that were characterised by all descriptive-fit statistics within the acceptable range. Among them, the four best-fitting models comprised indicators exclusively from either the realm of diagnosis questions or the forecasts. An indicator of the general economic situation was not present in either of the good fitting models. The two best fitting models were based on indicators regarding orders (IND.ORD), export orders (IND.EX.ORD), capacity utilisation (IND.CAP) with all the factor loadings being salient. The best fitting model comprised only states-related indicators: IND.ORD.S, IND.EX.ORD.S and IND.CAP.S (CFI = 0.996, TLI = 0.997, RMSEA = 0.039), and the second best model comprised the same set of questions but with regard to expectations: IND.ORD.F, IND.EX.ORD.F, and IND.CAP.F (CFI = 0.996, TLI = 0.997, RMSEA = 0.040).^24^ The fact that the set of indicators proves to be valid in both the coincident and leading version of the industrial confidence index provides strong support for the constant nature of interrelations between them and prompts acknowledgement of a common driving force behind these sets of questions. However, as the confidence indicator according to the definition presented in Sect. 2 should be future oriented, the most natural choice for a plausible indicator of industrial confidence (ICI) would be the one based on expectations. Therefore, the model results can be presented by the following system of equations:

4 $$ \left\{ {\begin{array}{*{20}l}    {IND.ORD.F^{t}  = 1 \cdot ICI^{t}  + \varepsilon _{1}^{t} \quad {\text{thresholds}}: - 0.357,1.007} \hfill  \\    {IND.EX.ORD.F^{t}  = \mathop {0.692}\limits_{{(0.032)}} {\text{ }} \cdot ICI^{t}  + \varepsilon _{2}^{t} \quad {\text{thresholds}}: - 0.471,0.848} \hfill  \\    {IND.CAP.F^{t}  = \mathop {0.746}\limits_{{(0.028)}}  \cdot ICI^{t}  + \varepsilon _{3}^{t} \quad {\text{thresholds}}: - 0.553,1.285} \hfill  \\   \end{array} } \right. $$

The results of model (4) should be interpreted as follows. A one-point increase in industrial confidence translates into a one-point increase on the scale of orders, a 0.692-point increase in the answers to the question regarding export orders and a 0.746-point increase in the response to question regarding capacity utilisation.

**Fig. 4:**
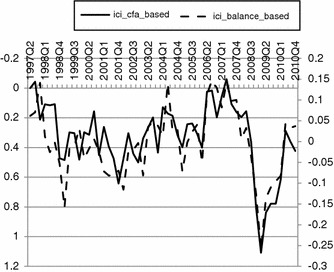
Confidence indicators calculated with the standard balance method and based on the confirmatory factor model. *Source*: Own calculations in Mplus 6.1 and SPSS 19

With a newly developed indicator in hand (ICI), we check whether the concept is significantly different (regarding correlations) from the concept of confidence based on the formative approach proposed by the European Commission (Fig. 4).

Although there are conceptual differences in the aggregation method between the confidence indicator obtained with confirmatory factor analytical approach and the indicator based on the standard European Commission methodology, their co-movement is clearly visible and supported by a correlation coefficient equal to −0.80.^25^ The differences in the forecasting validity of the constructs obtained with CFA and the standard method (with respect to the indicator of industrial production) are also hard to establish. The values of correlations between the indicators and the time series of interest—industrial production adjusted for the number of working days—are 0.291 and 0.214, respectively, for the indicator based on the methodology presented in the article and the one proposed by the European Commission. All of the relations have an expected sign, but only the correlation between the leading indicator of consumer confidence based on the MGCFA and industrial production is significant at the 0.05 level. However, on the other hand, the differences between the correlation coefficients with regard to the sample size—55 quarters—and thus, the power, are not significant (*p* value = 0.675), so it is not possible to reject the hypothesis of equal relation between each indicator and the industrial production dynamics. Thus, the results are not conclusive and require further research.

## Conclusions and Discussion

In this paper, we evaluated the current approach to measurement of industrial and consumer confidence. With application of Poland’s tendency survey data, we showed first that confidence indicators in the two most important tendency surveys—consumer and industrial—should not be treated as unidimensional. Second, we showed that operationalisation of both indices lacks consistency with respect to the construct validity and the industrial confidence indicator also lacks face validity. Surprisingly, all these deficiencies do not seem to limit the forecasting properties of the currently used indexes, which was shown in the study for the industrial confidence indicator.^26^

Nevertheless, we propose an alternative set of questions to calculate industrial confidence indicator and suggest analysing consumer confidence in its two dimensions. Our results, despite being not entirely conclusive, should in our opinion start a more in-depth discussion of the meaning of the confidence concept because, as shown in this paper, the term “confidence” is used in business and consumer tendency surveys without devoting much attention to the link between its definition and operationalisation. In the current approach to confidence measurement in tendency survey data, the issue of construct design was not taken into account when those indicators were first introduced. However, tendency surveys could greatly benefit from a systematic approach to constructing confidence indicators. Following the example of Wilcox et al. (2008) the confidence indicators would benefit from the reflective approach when used for forecasting purposes. The measurement in such a case could be based on indicators assessing either confidence, trust or beliefs of companies (households) that the economic situation will develop in a positive direction in a number of interrelated areas.

Despite the advances of the article, the approach we proposed has limitations. First, the conclusions were drawn based on the data for a single country; therefore, a more profound check for the validity of concepts of confidence should be conducted for other countries. Second, the results obtained in the field of consumer confidence rely strongly on the properties of the currently used set of consumer confidence items and the results obtained with respect to the industrial indicator are limited to models based on three items. With this in mind, an alternative conceptualisation may be considered and either a different set of questions for measuring consumer confidence can be proposed or a larger set of indicators of industrial confidence can be examined. A third limitation is the complexity of constructing indicators with multi-group confirmatory factor analysis. It requires individual data and is associated with re-estimation of the model when subsequent period is added.

As an alternative to reflective approach, a compromise with formative measurement might be used. In such a case the weighting scheme might be derived from a factor model or simply result from the principal component analysis (Nicoletti et al. 2000). This approach favours indicators with the largest correlation with the common factor variance and penalises those that are loosely related to it. Another approach, which puts an external variable of interest (and not the common variance) in the first place, is a regression-based approach. In this approach, the importance (weights) of items is estimated in a multivariate regression model (Sharpe and Andrews 2012). Additionally, this approach might be good in the case of confidence indicators from tendency surveys, as external variables are usually present in economic time series, which is rarely the case in psychological and sociological measurement.

This study shows also a potential for at least two very interesting areas of future research. First, more profound dimensionality analysis on business and consumer survey data can be performed, which might lead to establish relations between survey question responses. The established dimensions might be used in forecasting the main macroeconomic variables. Second, the results of consumer confidence indicate that consistency of responses decreases in periods when changes in the economic environment are present. These results need to be checked with larger, cross-country datasets to determine whether the forthcoming changes in consumer confidence are signalled by changing the pattern of response associated with decreasing goodness of fit statistics.

## Appendix 1

See Table 2.

**Table 2:** Set of questions with answers in the standardised consumer questionnaire

Question number and code	Question wording	Answer categories (representing also scale points)
Q1 (FS.S)	How has the financial situation of your household changed over the last 12 months? It has…	1.0 “got a lot better” 2.0 “got a little better” 3.0 “stayed the same” 4.0 “got a little worse” 5.0 “got a lot worse” −99 “do not know”
Q2 (FS.F)	How do you expect the financial position of your household to change over the next 12 months? It will…	1.0 “get a lot better” 2.0 “get a little better” 3.0 “stay the same” 4.0 “get a little worse” 5.0 “get a lot worse” −99 “do not know”
Q3 (GES.S)	How do you think the general economic situation in the country has changed over the past 12 months? It has…	1.0 “got a lot better” 2.0 “got a little better” 3.0 “stayed the same” 4.0 “got a little worse” 5.0 “got a lot worse” −99 “do not know”
Q4 (GES.F)	How do you expect the general economic situation in this country to develop over the next 12 months? It will…	1.0 “get a lot better” 2.0 “get a little better” 3.0 “stay the same” 4.0 “get a little worse” 5.0 “get a lot worse” −99 “do not know”
Q5 (PRA.S)	How do you think consumer prices have developed over the last 12 months? They have…	1.0 “risen a lot” 2.0 “risen moderately” 3.0 “risen slightly” 4.0 “stayed about the same” 5.0 “fallen” −99 “do not know”
Q6 (PRA.F)	By comparison with the past 12 months, how do you expect consumer prices will develop in the next 12 months? They will…	1.0 “increase more rapidly” 2.0 “increase at the same rate” 3.0 “increase at a slower rate” 4.0 “stay about the same” 5.0 “fall” −99 “do not know”
Q7 (UNEMP.F)	How do you expect the number of people unemployed in this country to change over the next 12 months? The number will…	1.0 “increase sharply” 2.0 “increase slightly” 3.0 “remain the same” 4.0 “fall slightly” 5.0 “fall sharply” −99 “do not know”
Q8 (MP.S)	In view of the general economic situation, do you think that now it is the right moment for people to make major purchases such as furniture, electrical/electronic devices, etc.?	1.0 “yes, it is the right moment now” 2.0 “it is neither the right moment nor the wrong moment” 3.0 “no, it is not the right moment now” −99 “do not know”
Q9 (MP.F)	Compared to the past 12 months, do you expect to spend more or less money on major purchases (furniture, electrical/electronic devices, etc.) over the next 12 months? I will spend…	1.0 “much more” 2.0 “a little more” 3.0 “about the same” 4.0 “a little less” 5.0 “much less” −99 “do not know”
Q10 (SAV.S)	In view of the general economic situation, do you think that now is…?	1.0 “a very good moment to save” 2.0 “a fairly good moment to save” 3.0 “not a good moment to save” 4.0 “a very bad moment to save” −99 “do not know”
Q11 (SAV.F)	Over the next 12 months, how likely is it that you save any money?	1.0 “very likely” 2.0 “fairly likely” 3.0 “not likely” 4.0 “not at all likely” −99 “do not know”
Q12 (FIN.S)	Which of these statements best describes the current financial situation of your household?	1.0 “we are saving a lot” 2.0 “we are saving a little” 3.0 “we are just managing to make ends meet on our income” 4.0 “we are having to draw on our savings” 5.0 “we are running into debt” −99 “do not know”

*Source*: European Economy (2006), The State of the Households Survey—Research Institute for Economic Development

## Appendix 2

See Table 3.

**Table 3:** Set of questions with answers in the standardised business tendency survey questionnaire

Question number and code	Question wording	Answer categories (representing also scale points)
Q1_S (IND.PROD.S)	Your production over the past month has…	+ increased = remained unchanged − decreased
Q1_F (IND.PROD.F)	Your production in the forthcoming 3–4 months…	+ will increase = will not change − will decrease
Q2_S (IND.ORD.S)	Your order books over the past month have been…	+ above normal = normal − below normal
Q2_F (IND.ORD.F)	Your order books in the forthcoming 3–4 months will be…	+ above normal = normal − below normal
Q3_S (IND.EX.ORD.S)	Your exports order books over the past month have been…	+ above normal = normal − below normal ? not applicable
Q3_F (IND.EX.ORD.F)	Your exports order books in the forthcoming 3–4 months will be…	+ above normal = normal − below normal ? not applicable
Q4_S (IND.STOCKS.S)	Your stocks over the past month have been…	+ above normal = normal − below normal
Q4_F (IND.STOCKS.F)	Your stocks in the forthcoming 3–4 months will be…	+ above normal = normal − below normal
Q5_S (IND.PRICES.S)	Your selling prices over the past month has…	+ increased = remained unchanged − decreased
Q5_F (IND.PRICES.F)	Your selling prices in the forthcoming 3–4 months…	+ will increase = will not change − will decrease
Q6_S (IND.EMPL.S)	Your firm’s total employment over the past month has…	+ increased = remained unchanged − decreased
Q6_F (IND.EMPL.F)	Your firm’s total employment in the forthcoming 3–4 months…	+ will increase = will not change − will decrease
Q7_S (IND.FS.S)	Your financial situation over the past month has…	+ improved = remained unchanged − deteriorated
Q7_F (IND.FS.F)	Your financial situation in the forthcoming 3–4 months…	+ will improve = will not change − will deteriorate
Q8_S (IND.GES.S)	The general economic situation (irrespectively of the situation of your branch and company) over the past month has…	+ improved = remained unchanged − deteriorated
Q8_F (IND.GES.F)	The general economic situation (irrespectively of the situation of your branch and company) in the forthcoming 3–4 months…	+ will improve = will not change − will deteriorate
Q9_S (IND.CAP.S)	Your capacity utilisation over the past month has…	+ increased = remained unchanged − decreased
Q9_F (IND.CAP.F)	Your capacity utilisation in the forthcoming 3–4 months…	+ will increase = will not change − will decrease

*Source*: European Economy (2006), Survey in the Manufacturing Sector—Research Institute for Economic Development

## Footnotes

1 Unidimensionality is required for reflective measurement; however, in the case of formative measurement, where runs from measures to construct (Diamantopoulos et al. 2008) it is also often assessed (OECD-JRC 2005; Saisana and Weziak-Bialowolska 2013).

2 However, we do not aim to conduct a full dimensionality analysis of all questions from the used surveys because it would require extensive data analysis and would change the focus of the paper away from confidence indicators.

3 The term “confidence” occurs in the European Commission document 170 times, and the formal link between the common-sense understanding of confidence and confidence as a business climate indicator is not provided (EuropeanCommission 2006), Additionally, there is no theoretical link stated between the confidence indicators from different areas. The only connection between confidence indicators from different areas is that they have their own, but also different, reference series. It should be assumed a flaw that does not blight the methodology developed in the common European methodology but needs to be improved to increase the communicability of this approach.

4 Which is a counterpart of consumer confidence in Europe.

5 Definition obtained from web page http://www.merriam-webster.com/dictionary/confidence on 6/8/2012.

6 Definition obtained from http://oxforddictionaries.com/definition/english/confidence on 6/8/2012.

7 Detailed wording of the questions used can be found in the Appendix 1 and 2.

8 This observation was also made and exploited with respect to the relation between stock market returns and confidence by Jansen and Nahuis (2003).

9 The formal differences between the two approaches to measurement can be found in Brown (2006).

10 This study is limited to the set of indicators that are currently present in tendency surveys and thus we do not exploit the possibility of creating a completely new instrument of confidence by developing a new set of indicators (questions). Such an approach would require redesign of the questionnaire with a focus on confidence, which was beyond the scope of the research.

11 Examples of such an approach are static and dynamic factor models.

12 A very informative summary of the research on measurement invariance with information about area and compared groups included can be found in Schmitt and Kuljanin (2008).

13 Arguments indicating that factor analysis or a more general latent variable model should be used to calculate the scores of concepts-by-postulation are provided by Saris and Gallhofer (2007:277–278).

14 It is usually the first element of this vector. Instead, by constraining one factor loading to 1, the identification of the measurement model can also be ensured by setting the variance of the latent variable to 1.

15 In Mplus, which was used for estimation purposes, categorical response variables are automatically recoded in such a way that the lowest category is labelled to 0.

16 It should be stated that two thresholds are predefined: $v_{p,0}^{t} = - \infty$ and $v_{{p,M_{p} + 1}}^{t} = + \infty$.

17 For further discussion on the issue of model fit, see Steenkamp and Baumgartner (1998), Hu and Bentler (1999), Marsh (2004) and Davidov (2008).

18 SMRM is not available in the WLSMV estimation procedure.

19 Quarterly data were used because the wording of the survey questions specifies that predictions made by companies are oriented 3–4 months ahead.

20 The procedure was of exploratory nature because there is not a single definition of the industrial confidence that hints on what indicators should be used.

21 Because not all of the answer categories were present in all periods for all of the questions (GES.F, UNEMP.F, FS.F and SAV.F), the most optimistic answer categories were combined with the second most optimistic ones.

22 In a model with two factors each explained by two items, there are no constraints that could be relaxed in order to test for partial measurement invariance (see condition for partial measurement invariance in the last paragraph of Sect. 3). Partial measurement invariance requires at least two indicators with fixed factor loading and thresholds and this condition in this specification is only fulfilled in the model with full measurement invariance.

23 Assessment of the configural invariance was not necessary. The model with three items and one group only is just identifiable and the correlations between items can be reproduced without error.

24 Although the number of estimated models was very large, due to very large datasets the confidence intervals of the fit statistics were very narrow and thus probability of obtaining the results by chance very low (i.e. in the two best models P(RMSEA > 0.06) <10^−9^).

25 The newly created ICI is inversely oriented due to this negative correlation was obtained.

26 The forecasting validity of consumer confidence was not subject to verification due to the lack of measurement invariance for the whole time–span, which resulted in shortening of the period of analysis to 17 quarters only.

## References

[CR1] Adams FG (1964). Consumer attitudes, buying plans, and purchases of durable goods: A principal components, time series approach. The Review of Economics and Statistics.

[CR2] Athanasoglou, S., Weziak-Bialowolska, D., & Saisana, M. (2014). *Environmental Performance Index 2014 JRC Analysis and Recommendations*—*JRC Science and Policy Reports* (Vol. Report EUR, p. 35). Luxembourg: European Union. doi:10.2788/64170.

[CR3] Baxter R (2009). Reflective and formative metrics of relationship value: A commentary essay. Journal of Business Research.

[CR4] Białowolski P, Kuszewski T, Witkowski B (2014). Bayesian averaging of classical estimates in forecasting macroeconomic indicators with application of business survey data. Empirica.

[CR5] Białowolski, P., & Węziak-Białowolska, D. (2013). The index of household financial condition, combining subjective and objective indicators: An appraisal of Italian households. *Social Indicators Research*. doi:10.1007/s11205-013-0401-0.

[CR6] Brown TA (2006). Confirmatory factor analysis for applied research.

[CR7] Browne, M. W., & Cudeck, R. (1993). Alternative ways of assessing model fit. In K. A. Bollen & J. S. Long (Eds.), *Testing structural equation models* (pp. 136–162). Newsbury Park, CA: Sage.

[CR8] Byrne BM, Shavelson RJ, Muthen B (1989). Testing for the equivalence of factor covariance and mean structures: The issue of partial measurement invariance. Psychological Bulletin.

[CR9] Byrne BM, van de Vijver FJR (2010). Testing for measurement and structural equivalence in large-scale cross-cultural studies: Addressing the Issue of nonequivalence. International Journal of Testing.

[CR10] Carroll CD, Fuhrer JC, Wilcox DW (1994). Does consumer sentiment forecast household spending? If so, why?. The American Economic Review.

[CR11] Chou C-P, Bentler PM, Hoyle RH (1995). Estimates and test in structural equation modelling. Structural equation modelling: Concepts, issues and applications.

[CR12] Coertjens L, Donche V, De Maeyer S, Vanthournout G, Van Petegem P (2012). Longitudinal measurement invariance of Likert-type strategy scales: Are we using the same ruler at each wave?. Journal of Psychoeducational Assessment.

[CR13] Coltman T, Devinney TM, Midgley DF, Venaik S (2008). Formative versus reflective measurement models: Two applications of formative measurement. Journal of Business Research.

[CR14] Costantini, M. (2013). Forecasting the industrial production using alternative factor models and business survey data. *Journal of Applied Statistics*, (July), 1–15. doi:10.1080/02664763.2013.809870.

[CR15] Curtin RT (1982). Indicators of consumer bahavior: The University of Michigan surveys of consumers. Public Opinion Quarterly.

[CR16] Davidov E (2008). A cross-country and cross-time comparison of the human values measurements with the second round of the European Social Survey. Survey Research Methods.

[CR17] De Jong MG, Steenkamp JEM, Fox J (2007). Relaxing measurement invariance in cross-national consumer research using a hierarchical IRT model. Journal of Consumer Research.

[CR18] Diamantopoulos A (2010). Reflective and formative metrics of relationship value: Response to Baxter’s commentary essay. Journal of Business Research.

[CR19] Diamantopoulos A, Riefler P, Roth KP (2008). Advancing formative measurement models. Journal of Business Research.

[CR20] European Commission. (2006). *European economy, special report no. 5, The Joint Harmonised EU Programme of Business and Consumer Surveys*.

[CR21] Gayer, C., & Genet, J. (2006). *Using factor models to construct composite indicators from BCS data*—*A comparison with European Commission Confidence Indicators* (No. 240).

[CR22] Golinelli R, Parigi G (2004). Consumer sentiment and economic activity: A cross country comparison. Journal of Business Cycle Measurement and Analysis.

[CR23] Horn JL, Mcardle JJ (1992). A practical and theoretical guide to measurement invariance in aging research. Experimental Aging Research: An International Journal Devoted to the Scientific Study of the Aging Process.

[CR24] Hox J (2002). Multilevel analysis. Techniques and applications.

[CR25] Hu L, Bentler PM (1998). Fit indices in covariance structure modeling: Sensitivity to underparameterized model misspecification. Psychological Methods.

[CR26] Hu L, Bentler PM (1999). Cutoff criteria for fit indexes in covariance structure analysis: Conventional criteria versus new alternatives. Structural Equation Modeling.

[CR27] Hurley AE, Scandura TA, Schriesheim CA, Brannick MT, Seers A, Vandenberg RJ, Williams LJ (1997). Exploratory and confirmatory factor analysis: Guidelines, issues, and alternatives. Journal of Organizational Behavior.

[CR28] Jansen WJ, Nahuis NJ (2003). The stock market and consumer confidence: European evidence. Economics Letters.

[CR29] Kaplan D (2009). Structural equation modeling. Foundation and extentions.

[CR30] Kumar V, Leone RP, Gaskins JN (1995). Aggregate and disaggregate sector forecasting using consumer confidence measures. International Journal of Forecasting.

[CR31] Lemmens A, Croux C, Dekimpe MG (2007). Consumer confidence in Europe: united in diversity. International Journal of Research in Marketing.

[CR32] Marsh HW (2004). In search of golden rules : Comment on hypothesis-testing approaches to setting cutoff values for fit indexes and dangers in overgeneralizing Hu and Bentler ’ s (1999) findings. Structural Equation Modeling.

[CR33] Matsunaga M (2011). How to factor-analyze your data right: Do’s, don’ts, and how-to’s. International Journal of Psychological Research.

[CR34] Meredith W (1993). MI, factor analysis and factorial invariance. Psychometrika.

[CR35] Meredith W, Teresi JA (2006). An essay on measurement and factorial invariance. Medical Care.

[CR36] Mueller E (1963). Ten years of consumer attitude surveys: Their forecasting record. Journal of the American Statistical Association.

[CR37] Muthen, B., & Asparouhov, T. (2002). Latent variable analysis with categorical outcomes: Multiple-group and growth modeling in Mplus. *Mplus Web Notes*, *4*. Retrieved from http://www.statmodel.com/download/webnotes/CatMGLong.pdf.

[CR38] Nahuis NJ, Jansen WJ (2004). Which survey indicators are useful for monitoring consumption? Evidence from European countries. Journal of Forecasting.

[CR39] Nicoletti, G., Scarpetta, S., & Boylaud, O. (2000).* Summary Indicators of Product Market Regulation with an Extension to Employment Protection Legislation*, OECD Economic Department Working Papers No. 226, OECD Publishing. doi:10.1787/215182844604.

[CR40] OECD-JRC. (2005). *Handbook on constructing composite indicators: Methodology and user guide*. doi:10.1787/533411815016.

[CR41] Osborne, J. W., & Costello, A. B. (2004). Sample size and subject to item ratio in principal components analysis. *Practical Assessment, Research & Evaluation*, *9*(11). Retrieved July 10, 2013 from http://PAREonline.net/getvn.asp?v=9&n=11.

[CR42] Paradiso, A., Kumar, S., & Margani, P. (2014). Are Italian consumer confidence adjustments asymmetric? A macroeconomic and psychological motives approach. *Journal of Economic Psychology*, 147–170. doi:10.1016/j.joep.2014.04.006.

[CR43] Pickering JF, Harrison JA, Cohen CD (1973). Identification and measurement of consumer confidence: Methodology and some preliminary results. Journal of the Royal Statistical Society. Series A.

[CR44] Raijman R, Davidov E, Schmidt P, Hochman O (2008). What does a nation owe non-citizens? National attachments, perception of threat and attitudes towards granting citizenship rights in a comparative perspective. International Journal of Comparative Sociology.

[CR45] Ramalho EA, Caleiro A, Dionfsio A (2011). Explaining consumer confidence in Portugal. Journal of Economic Psychology.

[CR46] Raykov T, Marcoulides GA, Cheng-Hsien L (2012). Measurement invariance for latent constructs in multiple populations: A critical view and refocus. Educational and Psychological Measurement.

[CR47] Saisana, M., & Weziak-Bialowolska, D. (2013). JRC statistical audit on the environment and gender index. In IUCN (Ed.), *The environment and gender index (EGI). 2013 Pilot*. Washington, DC.

[CR48] Saltelli A (2007). Composite Indicators between analysis and advocacy. Social Indicators Research.

[CR49] Saris WE, Gallhofer IN (2007). Design, evaluation, and analysis of questionnaires for survey research.

[CR50] Schmitt N, Kuljanin G (2008). Measurement invariance: Review of practice and implications. Human Resource Management Review.

[CR51] Shapiro HT, Angevine GE (1969). Consumer attitudes, buying intentions and expenditures: An analysis of the Canadian Data. The Canadian Journal of Economics.

[CR52] Sharpe, A., & Andrews, B. (2012). *An assessment of weighting methodologies for composite indicators: The case of the index of economic well*-*being*. Ottawa, Ontario. Retrieved from http://www.csls.ca/reports/csls2012-10.pdf.

[CR53] Steenkamp JEM, Baumgartner H (1998). Assessing measurement invariance in cross-national consumer research. The Journal of Consumer Research.

[CR54] Steinmetz H, Schmidt P, Wieczorek ATS, Schwartz SH (2007). Testing measurement invariance using multigroup CFA: Differences between educational groups in human values measurement. Quality & Quantity.

[CR55] Vandenberg RJ, Lance CE (2000). A review and synthesis of the measurement invariance literature: Suggestions, practices, and recommendations for organizational research. Organizational Research Methods.

[CR56] Vuchelen J (2004). Consumer sentiment and macroeconomic forecasts. Journal of Economic Psychology.

[CR57] Wilcox JB, Howell RD, Breivik E (2008). Questions about formative measurement. Journal of Business Research.

[CR58] Zarnowitz V (1992). Business cycles. Theory, history, indicators, and forecasting.

